# Metformin-Induced Lactic Acidosis: A Question of Time

**DOI:** 10.1155/2020/6962591

**Published:** 2020-10-21

**Authors:** Céline Bellefroid, Pierre Goffin, Julien Guntz, Marine Deville

**Affiliations:** ^1^Department of Anesthesia and Intensive Care, MontLegia Hospital, Groupe Santé CHC, Liège, Belgium; ^2^Laboratory of Clinical, Forensic, Industrial and Environmental Toxicology, Center for Interdisciplinary Research on Medicines, University Hospital of Liège, Liège, Belgium

## Abstract

Metformin is an oral antidiabetic largely prescribed in the treatment of type II diabetes. Overdose is associated with life-threatening lactic acidosis. We report the case of the highest metformin concentration ever described secondary to a voluntary suicidal intake. The patient developed a severe lactic acidosis and hemodynamic shock successfully treated with high-flow hemofiltration. Time to start extrarenal epuration is capital to avoid poor evolution.

## 1. Introduction

In 2014, metformin was the 7^th^ most frequently prescribed generic drug in the United States [1]. Metformin is a biguanide oral antidiabetic agent used as a first-line treatment of type II diabetes. Metformin decreases blood glucose levels by acting on intracellular insulin signal pathways, improving insulin sensitivity both by enhancing insulin-mediated suppression of gluconeogenesis and by reducing glucagon-stimulated gluconeogenesis [2].

Metformin toxicity is rare but may lead to severe acidosis with a mortality rate that can reach 50% [3].

In the therapeutic dose range, toxicity rarely occurs. There are only a few cases of metformin overdose in the literature. The present work illustrates a case of voluntary intake of very high doses of metformin, leading to severe lactic acidosis yet with a favorable outcome. Informed consent was obtained from the patient for the publication of this case report.

## 2. Clinical Case

A 23-year-old woman presented to the Emergency Department (ER) four hours after a suicide attempt by voluntary intake of 64 pills of metformin 850 mg (total intake of 54 g). She had a medical history of depression and type II diabetes mellitus.

**Figure 1:**
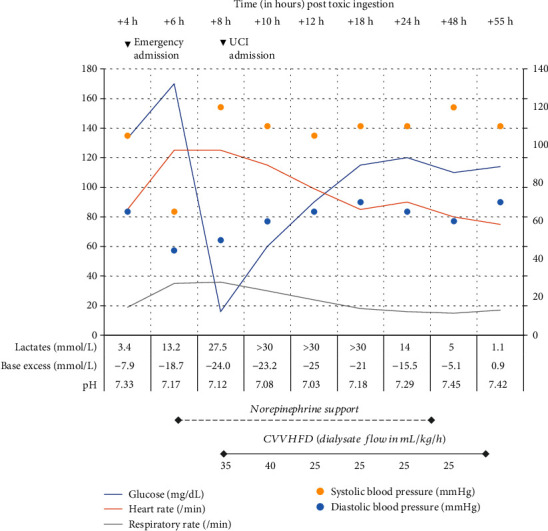
Physiological and biological parameters.

At admission, she was complaining of violent abdominal pain and anxiousness. She had severe polypnea. Anamnesis finally revealed the toxic ingestion. She progressively developed hypotension, and arterial blood gas analysis demonstrated lactic acidosis (Figure 1).

Supportive treatment was started at the ER. Hypotension was not corrected with fluid challenge (twice 500 mL boluses of crystalloid); therefore, vasoactive support was initiated (norepinephrine; maximum dose 0.18 *μ*g/kg/min). The patient was transferred to the intensive care unit (ICU) for initiation of continuous venovenous hemodiafiltration (CVVHDF) with regional citrate anticoagulation. Hemodialysis was not available at that moment. The total-to-ionized calcium ratio remained stable and inferior to 2.5 during filtration. Due to hypoglycemia, with the lowest level measured at 16 mg/dL, glucose infusion was started.

After more than 12 hours of CVVHDF (dialysate flow 25-40 mL/kg/h), normalization of arterial pH was nearly achieved and vasopressor administration could be discontinued (Figure 1).

Extrarenal epuration was then stopped after 48 hours. Three days after the patient's admission, glycemia and arterial blood analysis returned to normal values. The patient was authorized to leave the hospital after the psychologist's consent.

A few days after medical discharge, laboratory analyses showed that metformin serum concentration peaked at 660 mg/L 6 hours after intake and dropped at 2.90 mg/L 24 hours postingestion.

## 3. Discussion

Metformin overdose is relatively uncommon despite an annual estimate of 120 million prescriptions worldwide [4]. In chronic use and in therapeutic range, pharmacological studies demonstrated the highest detected metformin concentration being 5.5 mg/L [5]. In the therapeutic range, a systematic review of the literature showed an incidence of lactic acidosis ranging from less than 5.1 to 9 cases per 100,000 patients/year [6].

There are only a few cases of metformin overdose in the literature. The highest antemortem concentration reported did not exceed 380 mg/L [7], while it was 292 mg/L [8] in a patient who survived. The present case is of a survivor with the highest recorded concentration of metformin at 660 mg/L.

Metformin was quantified in serum by ultrahigh-performance liquid chromatography combined with a time-of-flight detector (UPLC-TOF-MS; Sciex). The method is further detailed in Supplementary Materials (available here).

Hyperlactatemia and metabolic acidosis are the hallmarks of biguanide toxicity. In physiologic conditions, skeletal muscle is a major source of lactate production through anaerobic glycolysis and the liver is largely responsible for lactate clearance through gluconeogenesis [9]. At therapeutic doses, metformin decreases liver gluconeogenesis without affecting skeletal muscle lactate release [10].

Metformin inhibits the mitochondrial form of glycerol-phosphate dehydrogenase (mDPD), leading to inhibition of hepatic gluconeogenesis, impairing mitochondrial function (inhibition of complex I) in the liver and other tissues and decreasing global oxygen consumption [11]. This reduction of cell redox function can lead to decreased lactate clearance through liver gluconeogenesis. A huge dose of metformin also increases skeletal muscle lactate production by impairing mitochondrial metabolism, leading to overproduction [12]. Diffuse inhibition of cellular respiration and secondary lactate overproduction may contribute to the development of metformin-induced lactic acidosis.

Metformin-induced change in the AMP/ATP ratio also activates AMPK, which suppresses lipid synthesis and is involved in the cardioprotective effects of metformin [13].

There are no specific symptoms associated with metformin-induced acute lactic acidosis. The clinical presentation is mainly affected by symptoms of mitochondrial dysfunction [11]: hypothermia, respiratory failure, hypotension, and eventually shock. Thus, medical diagnosis is based on clinical history. Despite severe hypoglycemia requiring temporal glucose infusion in the present case, hypoglycemia is not a major issue in case of overdose given the mechanism of action of metformin.

Metformin intoxication may temporarily decrease the levels of coagulation factors synthesized by the liver (coagulation factors II, V, VII, IX, and X) [14]. These factors were not assessed in blood; no hemorrhagic complication was observed.

From a pharmacologic point of view, metformin is mainly absorbed in the upper part of the intestine with a bioavailability of about 40-60%. The plasmatic peak level is quickly reached 1 to 2 hours after ingestion, and the plasmatic half-life is 4 to 8.7 hours. Metformin molecular weight is 165 Da with no plasma protein binding; it is not metabolized and is excreted unchanged through the renal route [15].

Aggressive therapy should be initiated as soon as possible. Extrarenal epuration is the first-line treatment, with the aim of decreasing metformin concentration and allowing the physiological lactate clearance. Indeed, endogenous lactate clearance is probably superior to hemofiltration [16].

Diffusive techniques as traditional hemodialysis are very efficient (approximate metformin clearance of 200 mL/min), whereas purely convective continuous techniques provide much lower metformin clearance (approximately 50 mL/min). Thus, the most appropriate method for emergency elimination is hemodialysis. Nevertheless, CVVH may be considered if hemodialysis is unavailable [17]. Although drug clearance by CVVH was less than generally reported by conventional dialysis, some authors did not find differences between both techniques [6]. Sustained low-efficiency dialysis (SLAD) is an intermittent hybrid renal replacement modality halfway between conventional intermittent hemodialysis and continuous renal replacement therapy. This prolonged diffusive technique may be a valuable option in case of hemodynamic instability [18]. After absorption, metformin is rapidly distributed in a tissue compartment with high volume distribution [19]. According to a two-compartment model, metformin shows a biphasic pattern of elimination. Therefore, it is necessary to proceed over a long filtration period due to recirculating metformin from an extraplasmatic compartment. In case of CVVH use, renal replacement therapy of over approximately 30 hours has been reported. Hemodialysis has the additional benefit of correcting the acidosis while removing toxins.

Even though cases report the use of regional citrate anticoagulation (RCA) in extracorporeal treatment for metformin intoxication without complication [18, 20], using RCA could be controversial.

Citrate is metabolized in the cytoplasm of hepatocytes by ATP citrate lyase which cleaves the citrate into acetyl-CoA and oxaloacetate in the cytoplasm [21]. This ATPase does not seem affected by metformin. Theoretically, the safety of citrate accumulation could be critical, especially in patients with liver failure. However, it is demonstrated that despite substantial accumulation of citrate in serum, RCA-CVVH seems feasible in patients with severely impaired liver function [22, 23]. It is important to identify patients at risk and assess careful monitoring of electrolyte status, especially calculating the calcium ratio [24].

Current recommendations from the Extracorporeal Treatments in Poisoning Workgroup do not contraindicate the use of regional citrate anticoagulation [17].

Second-line treatment is supportive care. Administration of vasopressor agents is mandatory to restore hemodynamic conditions. In some cases, the use of vasopressin demonstrated effectiveness [25]. Authors recommend the use of vasopressin when the patient is unresponsive to catecholamines [26].

There is no relevant correlation between metformin blood level and survival rate [27]. The outcome of metformin acute lactic acidosis is mainly influenced by the occurrence of multiple organ dysfunction and patient comorbidity [19]. The factor that seems crucial is the time to start extrarenal epuration which allows removal of metformin and correction of metabolic abnormalities. It is the only way to stop the vicious circle from lactic acidosis to multiple organ failure.

## 4. Conclusion

We present the case of a voluntary metformin intoxication resulting in the highest metformin concentration ever described. The patient's survival was due to a fast supportive treatment. It is capital to rapidly initiate extrarenal epuration in order to remove toxins and lead to acidosis correction. It is the only way to avoid fatal multiple organ failure due to major lactic acidosis.

## Supplementary Materials

Supplementary Materials Technical description of the metformin quantification method used in this case.
