# Supplementary material for: Metformin-Induced Lactic Acidosis: A Question of Time
Source: Case Rep Crit Care. 2020 Oct 21;2020:6962591. doi: 10.1155/2020/6962591 (PMC7596499; doi:10.1155/2020/6962591)
Supplement: Supplementary Materials — Technical description of the metformin quantification method used in this case. [file 6962591.f1.docx]

Supplementary Materials : Description of metformin quantification method.

Standards and chemicals. Analytical standards of metformin and metformin-d6 (used as internal standard) were purchased from LGC standards (Teddington, United Kingdom). All solvents were LC-MS or HPLC grade and supplied by J.T. Baker (Phillipsburg, USA). For mobile phases preparation, ammonium formate was supplied by Fisher Chemical (Merelbeke, Belgium) and formic acid was purchased from Biosolve (Dieuze, France).

Sample preparation. Metformin determination was performed on 200 µL of serum. After addition of 50 µL of 1 mg/L metformin-d6, a simple protein precipitation was done using 1 mL acetonitrile, which is then evaporated to dryness before reconstitution in the mobile phase and injection on UPLC-TOF-MS.

UPLC-TOF-MS analysis. The UPLC-TOF-MS apparatus was an Eksigent LC 100 XL combined with a TripleTOF 4600 from Sciex (Framingham, USA) and the screening method was developed by Sciex. Injections of 10 µl of the samples were done on a Kinetex C18 column, 2.6 µm, 100 Å, 50 x 3.00 mm (Phenomenex, Torrance, USA). A gradient was applied at 30°C, with mobile phase A consisting of 10 mM ammonium formate while mobile phase B was a mix of acetonitrile and methanol with 0.1% formic acid (50/50). A constant flow of 0.4 mL/min was applied using the following gradient: the initial condition of 98% of mobile phase A was held during 1 min. Then the gradient linearly decreased to 0 % of A in 10 min, held for 3 min. Finally, the gradient returned to initial conditions and was maintained for 2.5 min prior to the next injection. The TOF was equipped with a DuoSpray Ion source working in positive electrospray ionization (ESI) mode. Source conditions were as follows: ion source gas 1 (GS1) 40 psi, ion source gas 2 (GS2) 60 psi, curtain gas (CUR) 30 psi, source temperature 500°C and ion spray voltage floating 5500V. The mass acquisition method was a TOF survey scan from 50 to 1100 Da (cycle time 1 sec, accumulation time 0.150 sec), combined with a second experiment of product ion scan with an information dependent acquisition (IDA) method on a maximum of 20 candidates per cycle. Data were acquired with Analyst 1.7.1 software and finally processed with the MultiQuant software version 3.0.2.

Metformin quantification. Quantification was done using an external calibration. A working solution with a concentration of 10 mg/L of metformin was prepared in methanol. A ten fold dilution was done in methanol in order to obtain a diluted working solution with a concentration of 1 mg/L. Six calibrators were prepared by spiking blank matrix with an appropriate volume of working solution, in order to obtain metformin final concentrations of 0.100, 0.200, 0.400, 1, 1.50 and 2.00 mg/L.
